# New formulation and valid inequalities for a periodic capacitated vehicle routing problem with multiple depots, heterogeneous fleet, and hard time-windows

**DOI:** 10.1371/journal.pone.0335389

**Published:** 2025-10-31

**Authors:** Alejandro Arenas-Vasco, Juan Carlos Rivera, Maria Gulnara Baldoquín

**Affiliations:** Grupo de Investigación en Análítica y Cadenas de Suministro, Escuela de Ciencias Aplicadas e Ingeniería, Universidad EAFIT, Medellín, Antioquia, Colombia; Shanghai Jiao Tong University - Xuhui Campus, CHINA

## Abstract

This paper presents a new formulation and valid constraints for a *periodic capacitated vehicle routing problem with multiple depots, heterogeneous fleet, and hard time-windows* (MDHFPCVRP-TW). The problem raises from a real-world application in the vending machine industry in Medellín, Colombia. Our main contribution is a novel formulation that replaces binary depot-client assignment variables with continuous auxiliary variables and implements depot replication, achieving both model simplicity and computational efficiency. We introduce preprocessing techniques and valid constraints, particularly focusing on capacity-based constraints with client combinations, which significantly strengthen the formulation’s linear relaxation. Computational experiments demonstrate that our formulation consistently outperforms previous approaches across different instance sizes, achieving optimality for small instances and maintaining single-digit optimality gaps for medium-sized instances where earlier formulations showed gaps above 12%. The formulation shows particularly strong performance in solution time, often requiring less time to find feasible solutions. While limitations persist for very large instances, our results suggest promising directions for developing hybrid exact-heuristic methods for industrial-scale problems.

## Introduction

In Colombia, transportation represents over 30% of logistics costs for enterprises in the country, making efficient route planning crucial for profitability. The vending machine industry faces particular challenges, requiring periodic visits to maintain inventory levels and collect cash, while adhering to strict time windows and capacity constraints. This operational complexity, combined with Colombia’s urban geography and traffic conditions, makes optimization of delivery routes a critical factor for business sustainability.

The Vehicle Routing Problem (VRP) provides an ideal framework to model these real-world logistics challenges. By incorporating multiple attributes - periodicity (for weekly planning), multiple depots (reflecting distributed operations), heterogeneous fleet (accounting for different vehicle capacities), and hard time windows (meeting client schedules and company regulations) - the MDHFPCVRP-TW captures the essential characteristics of vending machine operations. The mathematical formulation of the described problems enables systematic optimization of route planning, potentially leading to significant cost reductions.

Valid inequalities and other cuts, and preprocessing techniques play a crucial role in strengthening mathematical formulations of complex routing problems. These enhancements tighten the linear relaxation bounds, reduce symmetry in the solution space, and eliminate unnecessary variables and constraints. For periodic vehicle routing problems (VRPs), where solution spaces grow exponentially depending on the number of nodes, such improvements can make the difference between finding optimal solutions (or even a feasible one) and computational intractability. Additionally, [1] demonstrated that the classical VRP is NP-hard. The MDHFPCVRP-TW is NP-hard since it can be reduced to the classical VRP by setting the number of depots to one, using a homogeneous fleet (single vehicle type), restricting to a single time period, and relaxing time window constraints to cover the entire planning horizon.

The proposed formulation, referred to hereafter as 3IFv2, represents a significant advancement in modeling MDHFPCVRP-TW. By replacing binary depot-client assignment variables with continuous auxiliary variables and implementing depot replication, it achieves a more elegant and computationally efficient representation of routing decisions. This novel approach, combined with carefully designed valid constraints and preprocessing techniques, enables the formulation to handle larger problem instances while achieving tighter bounds compared to previous approaches.

The remainder of this paper is organized as follows. We first present our proposed formulation for the MDHFPCVRP-TW, detailing the model’s sets, parameters, variables, and constraints. Next, we provide an overview of valid inequalities for Vehicle Routing Problems, focusing on classical constraints like the Dantzig-Fulkerson-Johnson subtour elimination constraints, comb inequalities, and multi-star inequalities. We then introduce a set of preprocessing techniques and valid constraints specifically designed to strengthen our formulation, including capacity-based constraints and pattern symmetry reduction techniques. The computational results section evaluates the impact of these enhancements on the model’s linear relaxation and compares the performance of our formulation against previous approaches across various instance sizes. Finally, we conclude by summarizing our findings and suggesting directions for future research, particularly regarding the development of solution methods for large-scale instances.

## Problem definition

### Statement of the problem

This paper aims at solving the MDHFPCVRP-TW problem presented in [2] using a novel formulation. The problem reflects a real-life scenario in the vending industry in Medellín, Colombia in which the objective is to plan routes a fleet of heterogeneous vehicles in a week (six days in this specific case) to minimize the total elapsed time fulfilling the clients’ demands. The final solution must indicate to the vending company how to route every vehicle each day in order to fulfill the clients’ demands.

Some fundamental considerations to take into account in this problem are:

- Although clients have hard time windows which are not day-dependent, vehicles are allowed to arrive before according to a maximum stand-by time allowed for each client. The service must end before the time window closes.
- Clients have some allowed delivery patterns indicating the specific days of the week in which demand can be delivered.
- Clients have a fixed demand in terms of vending machines. This demand is the same in every visit.
- Depots have a time window which comprehend the maximum duration of a route.
- Depots have a determined capacity in terms of number of vending machines that they can attend per day.
- Each vehicle operates under capacity constraints, limiting the number of vending machines it can service within a daily route. All routes must originate from and terminate at the same depot, forming a closed circuit.

### New mathematical formulation

In this new formulation, variables $x_{ij}^{h}$ take the value one if arc (i,j) ∈A is used on day *h* and zero otherwise (note that a vehicle index is not required). What differentiates it from the model 3IF presented in [2] is that instead of relying in binary variables like $r_{dc}^{h}$ and $w_{i}^{kh}$ to assign a node and a depot to a vehicle, we use continuous variables ${ℵ}_{ij}^{h}$ to do it. For this formulation to work, we replicate every depot such that we have a depot for each type of vehicle that can be used. This allow us to know which vehicle type performs a route. Then we add a parameter $\phi_{d}$ for each original depot. Using and relating variable ${ℵ}_{ij}^{h}$ and parameter $\phi_{d}$ we know which depot served every client and guarantee that each vehicle returns to the depot they departed at the end of the route. This formulation works for any quantity of periods even tough here a six-day week is considered.

The formulations use the following sets:

- *C* is the set of clients, where |C|=n.
- *D* is the set of depots (including replicated depots).
- *N* is the set of all nodes, conformed by clients and depots (N=C∪D).
- *F* is the set of possible frequencies of visit.
- *CP*_*f*_ is the set of clients with frequency *f*, where $C=\cup_{f\in F}CP_{f}$ and $\cap_{f\in F}CP_{f}=\emptyset$.
- *K* is the set of types of vehicles.
- *H* is the set of days. As referenced before, the scheduling span is of 6 days in this research.
- Directed complete graph *G*(*A*,*N*) where A={(i,j) | i,j ∈ N} is the set of arcs.
- *P*_*f*_ is the set of possible patterns of visit of frequency *f*.
- *DD*_*d*_ is the set of depots replicated from depot *d* plus itself.
- *DK*_*k*_ is the set of depots assigned to type of vehicle *k*.

Let us illustrate sets *DD*_*d*_ and *DK*_*k*_ using an example: if the problem has two depots $\left\{d_{1},d_{2}\right\}$ and two types of vehicles $\left\{v_{1},v_{2}\right\}$, depot *d*_1_ has a replica $d_{1^{\prime }}$ for vehicle type $v_{2}$, and depot *d*_2_ has a replica $d_{2^{\prime }}$ for vehicle type $v_{2}$. Then we have, set $D=\{d_{1},d_{1^{\prime }},d_{2},d_{2^{\prime }}\}$, set $DK_{1}=\{d_{1},d_{2}\}$, set $DK_{2}=\{d_{1^{\prime }},d_{2^{\prime }}\}$, set $DD_{1}=\{d_{1},d_{1^{\prime }}\}$, and $DD_{2}=\{d_{2},d_{2^{\prime }}\}$.

The formulation uses the following parameters:

- *q*_*k*_ is the capacity of the vehicle of type *k* as a function of the number of vending machines that it can attend daily.
- *q*${\;\;}^{\text{m}}$ is the maximum capacity among available types of vehicles.
- *dem*_*c*_ is the number of vending machines of client *c* that must be supplied in each visit.
- *s*_*c*_ is the time to serve client *c*.
- $\left[a_{i},b_{i}\right]$ is the time window in which node *i* must be attended (the upper bound for arriving at a depot is the maximum time duration allowed for a route). All the depots share the same upper bound.
- *l*_*c*_ is the maximum stand-by time allowed in client *c*.
- *t*_*ij*_ is the time to traverse arc (*i*,*j*) with any vehicle.
- *R*_*d*_ is the number of vending machines that depot *d* can attend daily.
- $veh_{k}$ is the number of vehicles available of type *k*.
- $vis_{f}$ is the number of visits that must receive a client with frequency *f* in the planning horizon.
- $A_{p}^{h}$ is an element of matrix *A*, which relates patterns with the days a client is visited. It takes the value 1 if pattern *p* forces clients to be visited on day *h* and 0 otherwise.
- A largely enough value (**M**) is used. According to the types of restrictions where M is used, it could be taken as the upper bound of time window of the depots.
- $\phi_{d}$ is a positive natural number assigned to depot *d*. It is used in the model’s auxiliary variable to relate the route of some vehicle that departed from *d*. $\phi_{d}$ is different for every element of set *D*.
- $\phi^{m}$ is the maximum value of $\phi_{d}$.
- *tmin*_*c*_ is the minimum distance from client *c* to any depot.

The complete model is now presented. Let us start with the variables:

- $x_{ij}^{h}\in \{0,1\}$: binary decision variable which takes the value 1 if the arc (*i*,*j*) is used in the day *h*, and 0 otherwise.
- $u_{cp}\in \{0,1\}$: binary decision variable which takes the value 1 if pattern *p* is assigned to client *c*, and zero otherwise.
- $T_{ij}^{\;h}\geq 0$: time at which a vehicle arrives to node *j* coming from node *i* on day *h*. Each vehicle can depart from any depot starting after the opening of the time window of such depot.
- $f_{ij}^{h}\geq 0$: load of the vehicle when it is traversing arc (*i*,*j*) on day *h*.
- ${ℵ}_{ij}^{\;h}\geq 0$: auxiliary decision variable to know if a route is assigned to a depot. It takes the value of $\phi_{d}$ if the arc (*i*,*j*) is served with a vehicle that departed from *d*, and zero otherwise.
- $y_{c}^{h}\geq 0$: stand-by time of the vehicle visiting client *c* on day *h*.

Model (1) to (27) presents the 3IF version 2 (3IFv2).

$$\text{min}\;Z=\sum_{h\in H}\sum_{i\in N}\sum_{\overset{j\in N}{i\neq j}}((t_{ij}+s_{j})\cdot x_{ij}^{h})+\sum_{c\in C}\sum_{h\in H}y_{c}^{h}$$ (1)

$$\text{s.t.}\;\sum_{h\in H}\sum_{\overset{i\in N}{i\neq c}}x_{ic}^{h}=vis_{f},\;\forall \;f\in F,\;c\in CP_{f}$$ (2)

$$\sum_{p\in P_{f}}u_{cp}=1,\;\forall \;f\in F,\;c\in CP_{f}$$ (3)

$$\sum_{\overset{i\in N}{i\neq c}}x_{ic}^{h}=\sum_{p\in P_{f}}A_{p}^{h}\cdot u_{cp},\;\forall \;f\in F,\;c\in CP_{f},\;h\in H$$ (4)

$$\sum_{\overset{i\in N}{i\neq c}}x_{ic}^{h}-\sum_{\overset{j\in N}{j\neq c}}x_{cj}^{h}=0,\;\forall \;c\in C,\;h\in H$$ (5)

$$\sum_{\overset{i\in N}{i\neq c}}T_{ic}^{h}+y_{c}^{h}\geq a_{c}\cdot \sum_{\overset{i\in N}{i\neq c}}x_{ic}^{h},\;\forall \;c\in C,\;h\in H$$ (6)

$$\sum_{\overset{i\in N}{i\neq c}}T_{ic}^{h}+y_{c}^{h}\leq (b_{c}-s_{c})\cdot \sum_{\overset{i\in N}{i\neq c}}x_{ic}^{h},\;\forall \;c\in C,\;h\in H$$ (7)

$$y_{c}^{h}\leq l_{c},\;\forall \;c\in C,\;h\in H$$ (8)

$$\sum_{\overset{i\in N}{i\neq c}}T_{ic}^{h}+y_{c}^{h}+s_{c}+t_{cj}-M\cdot (1-x_{cj}^{h})\leq T_{cj}^{h},\;\forall \;c\in C,\;h\in H,\;j\in N,\;c\neq j$$ (9)

$$\sum_{\overset{i\in N}{i\neq c}}T_{ic}^{h}+y_{c}^{h}+s_{c}+t_{cj}+M\cdot (1-x_{cj}^{h})\geq T_{cj}^{h},\;\forall \;c\in C,\;h\in H,\;j\in N,\;c\neq j$$ (10)

$$T_{dc}^{h}\geq t_{dc}\cdot x_{dc}^{h},\;\forall \;c\in C,\;d\in D,\;h\in H$$ (11)

$$T_{dc}^{h}\leq (b_{c}-s_{c})\cdot x_{dc}^{h},\;\forall \;c\in C,\;d\in D,\;h\in H$$ (12)

$$\sum_{\overset{i\in N}{i\neq c}}(f_{ic}^{\;h}-f_{ci}^{\;h})=dem_{c}\cdot \sum_{\overset{j\in N}{j\neq c}}x_{cj}^{h},\;\forall \;c\in C,\;h\in H$$ (13)

$$\sum_{d\in DK_{k}}\sum_{j\in C}x_{dj}^{h}\leq veh_{k},\;\forall \;k\in K,\;h\in H$$ (14)

$$f_{dj}^{\;h}\leq q_{k}\cdot x_{dj}^{h},\;\forall \;k\in K,\;d\in DK_{k},\;h\in H,\;j\in C$$ (15)

$$\sum_{d^{\prime }\in DD_{d}}\sum_{j\in C}f_{d^{\prime }j}^{\;h}\leq R_{d},\;\forall \;d\in D,\;h\in H$$ (16)

$$\sum_{j\in C}x_{dj}^{h}=\sum_{i\in C}x_{id}^{h},\;\forall \;d\in D,\;h\in H$$ (17)

$${ℵ}_{ij}^{h}\leq \phi^{m}\cdot x_{ij}^{h},\;\forall \;i\in C,\;j\in C,\;i\neq j,\;h\in H$$ (18)

$${ℵ}_{dj}^{h}=\phi_{d}\cdot x_{dj}^{h},\;\forall \;d\in D,\;j\in C,\;h\in H$$ (19)

$${ℵ}_{jd}^{h}=\phi_{d}\cdot x_{jd}^{h},\;\forall \;d\in D,\;j\in C,\;h\in H$$ (20)

$$\sum_{\overset{i\in N}{i\neq j}}({ℵ}_{ij}^{h}-{ℵ}_{ji}^{h})=0,\;\forall \;j\in C,\;h\in H$$ (21)

$$x_{ij}^{h}\in \{0,1\},\;\forall \;h\in H,\;i,j\in N,i\neq j$$ (22)

$$u_{cp}\in \{0,1\},\;\forall \;f\in F,\;c\in CP_{f},\;p\in P_{f}$$ (23)

$$f_{ij}^{\;h}\geq 0,\;\forall \;i\in N,\;j\in N,\;i\neq j,\;h\in H$$ (24)

$$0\leq T_{ic}^{h}\leq (b_{c}-s_{c}-tmin_{c}),\;\forall \;c\in C,\;h\in H,\;i\in N,\;i\neq c$$ (25)

$$y_{i}^{h}\geq 0,\;\forall \;h\in H,\;i\in C$$ (26)

$${ℵ}_{ij}^{h}\geq 0,\;\forall \;i\in N,\;j\in N,\;i\neq j,\;h\in H$$ (27)

The objective function (1) minimizes the total time of all routes, specifically, it considers traveling times, service times, and stand-by times. As the operating cost of the enterprise is principally based on time spent on a route, all times are given the same importance.

Constraints (2) to (4) define that every client is visited the number of times that their frequency dictates with a feasible pattern: (2) define the number of visits of every client, (3) guarantee that each client has an assigned pattern of visit, (4) force one route to visit a client each day the chosen pattern indicates it must be visited. Constraints (5) ensure that every client on any day is left after being visited. Constraints (6) to (8) condition the arrival time of the vehicles in the time window of every client, and the stand-by time of the vehicles: (6) and (7) require that each visited client respect the time window, considering the service time and the possible stand-by time, while (8) force the vehicles on any day to comply with the maximum stand-by time allowed in every client. Constraints (9) to (12) condition the time of arrival to every node. Constraints (9) and (10) allow to compute, when $x_{cj}^{h}=1$, the arrival time to node *j* coming from node *c* considering arrival time to node *c*, traveling time, service time and stand-by time. They become inactive when $x_{cj}^{h}=0$. (11) guarantee that the arrival time to the first client from a depot considers the time required to travel from the depot to this first client. (12) force the arrival time to a client from a depot to be zero if the client is not visited directly from that depot. Note that vehicles are not forced to leave the depot on time zero. Constraints (13) indicate that the difference between load in and load out at a node must be equal to the node’s demand.

Constraints (14) guarantee that the number of used vehicles does not exceed the number of vehicles available of each type. Constraints (15) do not allow the load of used vehicles to exceed its capacity. Constraints (16) force the accumulated load departing from every depot to be limited by its capacity. Constraints (17) limit the number of vehicles that depart from a depot to be equal to the number of vehicles that arrive at such depot. Constraints (18) to (21) assign each route to a depot: (18) force the auxiliary variable that relates an arc with a depot to be zero if such arc is not used, (19) guarantee that each route departing from a depot it assigned to it, (20) make each route to arrive to its origin depot, and (21) are the flow constraints.

Constraints (22) to (27) indicate the domain of the decision variables.

The main difference between 3IF and 3IFv2 lies in how routes are assigned to depots and tracked in the model. While 3IF uses binary variables $r_{dc}^{h}$ and $w_{i}^{kh}$ to explicitly assign nodes and depots to clients and trace vehicle usage, 3IFv2 employs a different approach with continuous auxiliary variables ${ℵ}_{ij}^{h}$ and depot replication. This transformation allows 3IFv2 to naturally capture vehicle-depot assignments through the model’s structure: each depot is replicated for each vehicle type, and the auxiliary variable ${ℵ}_{ij}^{h}$ takes the value $\phi_{d}$ when an arc is served by a vehicle from depot *d*, effectively tracking depot assignments and route continuity. Beyond simplifying the variable structure, this approach reduces the total number of binary variables in the model while maintaining its ability to enforce routing constraints. Additionally, 3IFv2 introduces parameter $\phi_{d}$ and its maximum value $\phi^{m}$, which work in conjunction with the auxiliary variables to ensure proper route construction and depot assignments, providing a more streamlined way to model the routing decisions compared to the explicit assignment approach of 3IF.

**Fig 1:**
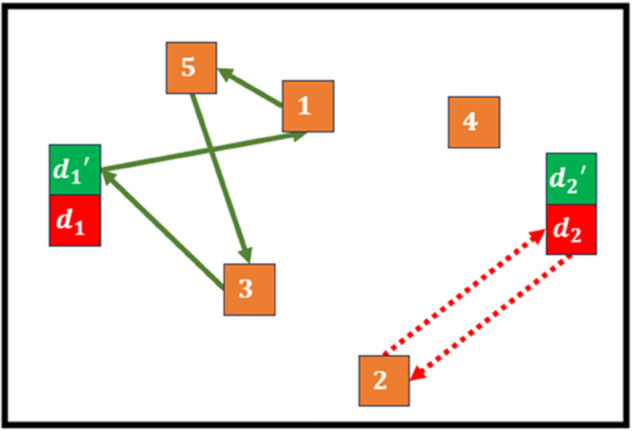
Example to understand the usage of variables ${ℵ}_{ij}^{h}$.

The inclusion of variables ${ℵ}_{ij}^{h}$ is the most important concept of this paper. As a consequence, an example of its usage is presented in Fig 1. In this example, index *h* is dropped because the relations of the variable are the same for each day. Let us consider a graph with five clients (1 to 5), two depots (*d*_1_ and *d*_2_ with assigned $\phi_{1}$ and $\phi_{2}$ respectively), and two types of vehicles ($v_{1}$ is represented by discontinuous lines and $v_{2}$ by continuous lines). Because there are two types of vehicles, each depot have to be replicated once (hence we have depots $d_{1}^{\prime }$ and $d_{2}^{\prime }$ for $v_{2}$; and the others for $v_{1}$).

Note that $v_{1}$’s route is $d_{2}\to 2\to d_{2}$. Meanwhile, $v_{2}$’s route is $d_{1}^{\prime }\to 1\to 5\to 3\to d_{1}^{\prime }$. Variables *x*_*ij*_ and ${ℵ}_{ij}$ for that particular day are represented as:



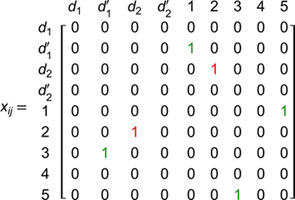





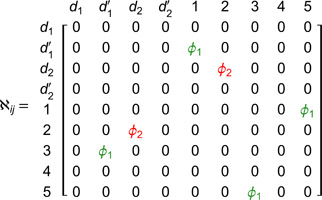



Note that ${ℵ}_{ij}$ lets the formulation know that a vehicle of type $v_{1}$ departed depot two (${ℵ}_{d_{2},2}=\phi_{2}$), and a vehicle of type $v_{2}$ departed depot one (${ℵ}_{d_{1}^{\prime },2}=\phi_{1}$).

## Overview of valid inequalities for VRP

**Fig 2:**
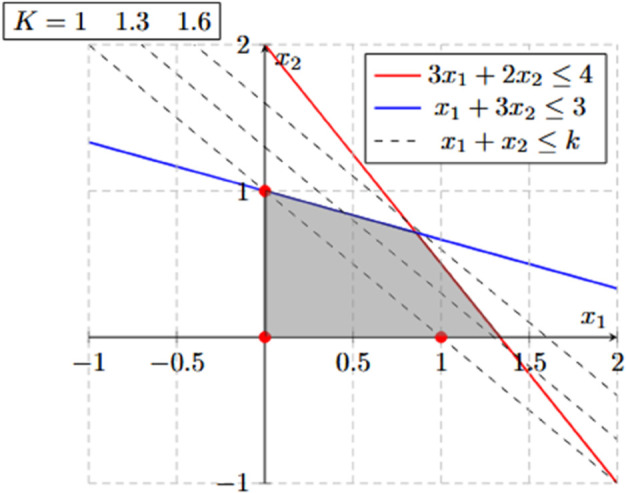
Feasible region of a MILP with the dotted lines representing the limit of three valid inequalities.

A valid inequality for a mixed integer linear programming (MILP) is a constraint that can be added to the formulation and does not eliminate any feasible integer solution. According to [3] a strong valid inequality (or cutting plane) is a constraint that, when added to the MILP, it improves the bound obtained using the linear relaxation. Fig 2 presents the feasible region of the linear relaxation of a MILP, which is limited by constraints $3x_{1}\;+\;2x_{2}\leq 4$ and $x_{1}\;+\;3x_{2}\leq 3$. Variables *x*_1_ and *x*_2_ are positive integers. A valid inequality for the problem is $x_{1}\;+\;x_{2}\leq k$ with k≥1. The valid inequality is better or worse depending on *k*’s value. For instance, with *k* = 1.6, the valid inequality is not useful because it is redundant. If *k* = 1.3, the valid inequality is useful as it eliminates part of the feasible region of the linear relaxation without eliminating any feasible integer solution. Finally, the valid inequality with *k* = 1 is the best option because it makes the vertexes of the new feasible region integers.

Before detailing some classical valid inequalities used in VRPs, it is paramount to clarify that the considered graph meets the conditions of the one described in the previous section. This is because when time windows are considered, the direction in which an arc is traversed is important.

Let us introduce some additional notation. Being τ⊆A, we define $x(\tau ):=\sum_{(i,j)\in \tau }x_{ij}$. For $S_{1},S_{2}\subseteq N$, we define $x(S_{1},S_{2})=\sum_{i\in S_{1}}\sum_{\begin{array}{c} j\in S_{2}(i,j)\in A \end{array}}x_{ij}$. For S⊆N, we define x(γ(S)):=x(S,S), $x(\delta^{+}(S)):=x(S,N⧵S)$ and $x(\delta^{-}(S)):=x(N⧵S,S)$. This implies that $\delta^{+}(S)$ are all the outgoing arcs of *S*. Meanwhile, all the incoming arcs of *S* are $\delta^{-}(S)$. As a consequence, $x(\delta^{+}(S))$ and $x(\delta^{-}(S))$ are their *x*_*ij*_ variables. For i∈N, we write $\delta^{+}(i)$ and $\delta^{-}(i)$ instead of $\delta^{+}(\{i\})$ and $\delta^{-}(\{i\})$ to simplify notation.

In this section some of the most known inequalities for VRPs are presented. There is a myriad of valid inequalities in the topic, and covering all of them is beyond the scope of this article. The reader is referred to [4] for a wider study of valid inequalities for VRPs.

### Dantzig, Fulkerson, and Johnson subtour elimination constraints

**Fig 3:**
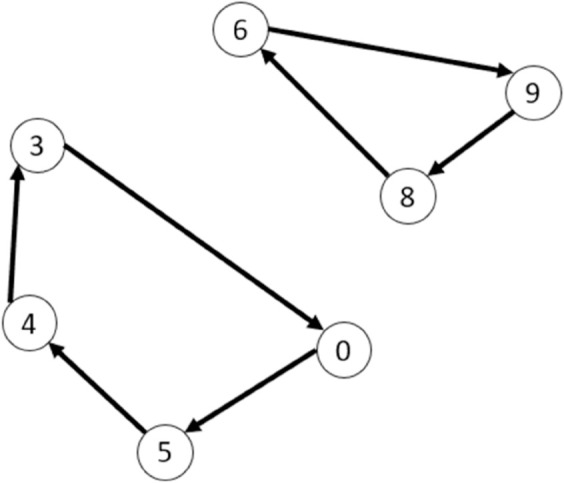
Example of a subtour.

The classical VRP has three main constraints that must be taken into account: the routes must start and come back to the depot, all clients must be served, and vehicles must leave any clients that they visit. The problem is that these three conditions do not always result in one Hamiltonian cycle. In Fig 3, a solution considering the mentioned constraints is shown. In this solution, the vehicle starts and returns to the depot, and the vehicle leaves all visited clients. But this solution is not feasible because it does not represent a Hamiltonian cycle.

Eliminating subtours efficiently has been an important task for operations research practitioners since the TSP was proposed. There are some alternatives to do so, and they are described in [5]. One of these alternatives could be added as valid inequalities iteratively. Proposed by [6], it is usually abbreviated as the DFJ constraints (as they were proposed by Dantzig, Fulkerson, and Johnson). These are the DFJ constraints:


x(γ(S))≤|S|−1  S∈N, 2≤|S|≤|N|

Note that, if S:={6,8,9} the solution in Fig 3 is no longer feasible as x(γ(S))=3 and |S|−1=2.

The problem with the DFJ constraints is that adding all of them is not practical for large instances. This is because they are exponential. But, adding them iteratively strengthens the linear relaxation of the MILP, so they are often used as valid inequalities.

### Comb inequalities

**Fig 4:**
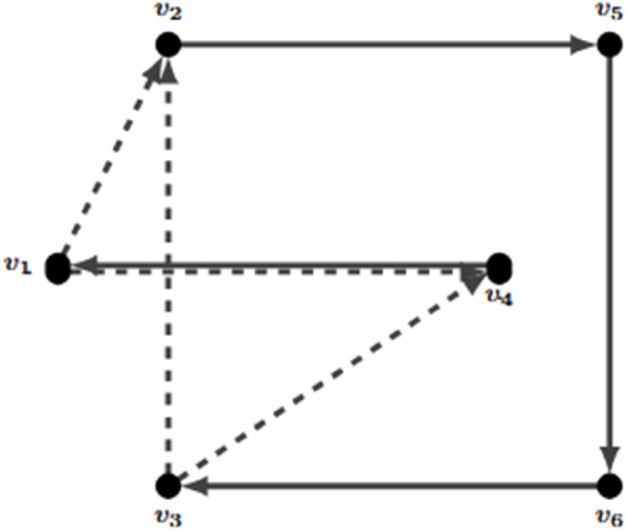
TSP’s graph representing an infeasible solution.

The comb inequalities were introduced to the TSP by [7]. These inequalities make the problem’s linear relaxation tighter. Let us present an example. The graph in Fig 4 represents the solution of a linear relaxation of a TSP.

**Fig 5:**
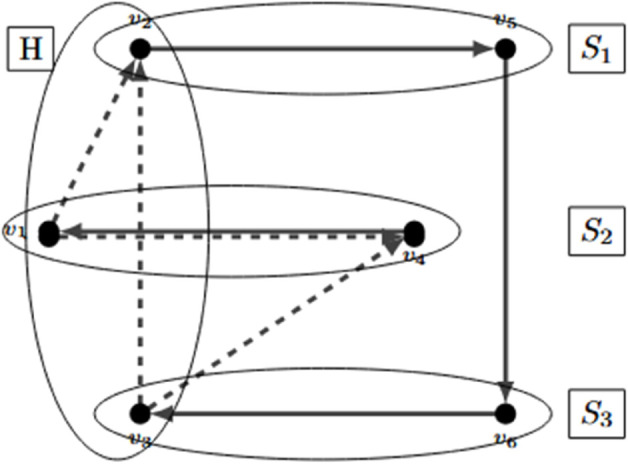
Design of a comb over a graph.

The weight of arcs in the graph are the optimal values of the *x* variables. Continues lines represent arcs with value 1, while dashed ones represent arcs which variables are fractional. But as a linear relaxation, it works perfectly as every node receives one unit of flow and delivers one unit of flow. To break this infeasible solution, a comb inequality can be added to the linear relaxation of the problem. A comb consists of a node-set *H* (which is called the handle) and node sets $S_{1},...,S_{p}$ (the teeth) such that: p≥3 and odd. Also, $H\cap S_{j}$ and $S_{j}⧵H$ are not empty for all *j*. In Fig 5 a comb is presented for the example with *p* = 3. In this case, H:={1,2,3}, *S*_1_ := {2,5}, *S*_2_ := {1,4}, and *S*_3_ := {3,6}.

The comb inequalities for directed graphs are formulated as follows:


$$x(\delta^{+}(H))+\sum_{j=1}^{p}x(\delta^{+}(S_{j}))\geq \frac{3p+1}{2}$$

This inequality breaks the previously presented infeasible solution. Let us break the inequality step-by-step: $x(\delta^{+}(H))=x_{41}\;+\;x_{63}$, $x(\delta^{+}(S_{1}))=x_{12}\;+\;x_{32}$, $x(\delta^{+}(S_{2}))=x_{34}$, and $x(\delta^{+}(S_{3}))=x_{56}$. So, the left side of the comb inequality for the non-feasible solution is $x_{41}+x_{63}+x_{12}+x_{32}+x_{34}+x_{56}=1+1+\frac{1}{2}+\frac{1}{2}+\frac{1}{2}+1=4.5$. The right side with *p* = 3 is 5, which is greater than 4.5. This comb inequality cuts the infeasible TSP solution from the linear relaxation.

[8] presented how the comb inequalities could be used in a VRP within a directed graph. The comb inequality holds, but the depot must be included in the handle.

### Multi-star inequalities

The Multi-star inequalities define a group of valid inequalities that consider the maximum number of arcs that can be assigned to a route. Let us define *Q* as such a number (*Q* can be obtained considering the capacity of the vehicles, the demand of the clients or other attributes like vehicle autonomy, route length limit, depot capacity). The multi-star inequalities were proposed by [9] for the undirected VRP. Later, [10] presented the multi-star inequalities for directed VRPs.

The multi-star inequalities require two disjoint sets of nodes (*S*_1_ and *S*_2_) that do not include the depot. A multi-start inequality could be written as follows:


$$Qx(S_{1})+x(S_{1},S_{2})+x(S_{2},S_{1})\leq (Q-1)|S_{1}|$$

**Fig 6:**
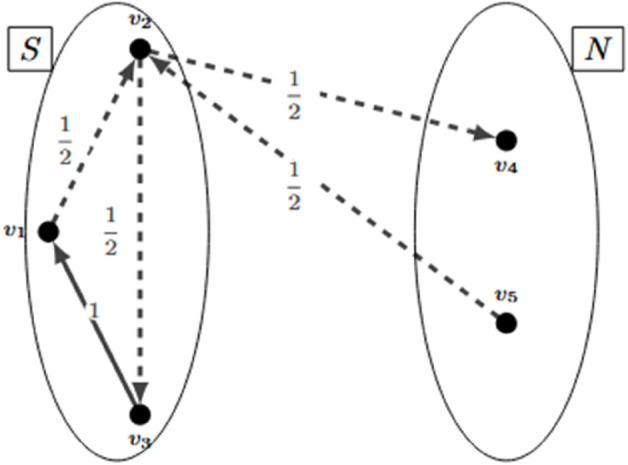
Example of a multi-star inequality.

Let us present an example. In Fig 6, a graph with five clients is presented (it represents a partial solution, other nodes are not depicted). Set *S*_1_ has clients one, two, and three, and set *S*_2_ has the remaining clients. Let *Q* = 3. A fractional solution is presented. Note that the sum of all the used arcs totals three, complying with the value of *Q*. A multi-star inequality breaks this fractional solution because $x(S_{1})=x_{12}+x_{23}+x_{31}=2$, $x(S_{2},S_{1})=x_{52}=\frac{1}{2}$, and $x(S_{1},S_{2})=x_{24}=\frac{1}{2}$. This results in the left side of the inequality being $3(2)+\frac{1}{2}+\frac{1}{2}=7$, which is greater than the right side ((3−1)3=6).

Unfortunately, using these valid inequalities were challenging in the previous formulation. The reason is simple: due to variables *u*_*cp*_ conditioning how clients are served in the different days of the problem, we do not have a notion on which arcs can be used. We tried using them only with clients that must be served everyday, but the results indicated no improvement in the performance of the solver. If a matheuristic approach decides when clients must be served first, and then the routing, all of these valid inequalities could be used. As a consequence, in the following section, we present a mix of valid inequalities and equalities, and preprocessing techniques to strengthen the linear relaxation of the MDHFPCVRP-TW.

### Valid inequalities and formulations for the PVRP

The PVRP was first studied in 1974 [11], motivated by waste collection challenges. However, the term “period routing problem” was not coined until ten years later [12]. According to [13], prior to 2010, the literature on exact methods applied to the PVRP remained limited. [13] proposed an exact method for solving the PVRP using four distinct classes of lower bounds based on problem reduction through constraint elimination, followed by the incorporation of valid inequalities from the CVRP (particularly generalized capacity constraints and clique cuts).

In [14], the authors presented seven valid inequalities for the PVRP with due dates, adapting these inequalities to three different formulations: flow based, flow based with assignment variables, and load based.

In the load based formulation, variables $x_{ij}^{h}$ serve the same purpose as in our formulation. It requires an additional set of variables $f_{ij}^{h}$ which indicate the load of a vehicle upon traversing arc (*i*,*j*) on day *h* as in our formulation. As a consequence, we can consider the load based formulation in [14] as a similar formulation to the one presented in this paper. The authors presented seven inequalities which were based on the demand served in a subset $H^{\prime }\subset H$, or in the number of used arcs to guarantee that the demand is met. We did not try these type of valid inequalities because in our formulation it is not possible to know when a client is visited beforehand.

The other two formulations proposed in [14] had the following characteristics: the flow based formulation used binary variables $x_{ijk}^{h}$ to indicate if vehicle *k* traversed arc (*i*,*j*) on day *h*; it resembles the 4IF that we presented previously on [2]. The flow based formulation with assignment variables included binary variables $z_{ik}^{h}$ which indicated if client *i* was visited by vehicle *k* on day *h*; it resembled the 3IF that we presented previously on [2].

Even though [13] in 2010 opened the door for the usage of more exact-driven methods to solve the PVRP, the bibliography still lacks depth. Additionally, most of the used valid inequalities for the PVRP are adaptations of valid inequalities for the CVRP which require the knowledge of the time period in which each client is served. Our formulation does not comply with this characteristic. As a consequence, we decided to proposed our own set of valid constraints and preprocessing techniques for our version of the problem.

## Proposed valid constraints and preprocessing for the MDHFPCVRP-TW

This section presents multiple strategies to make the MDHFPCVRP-TW more efficient. Strategies vary from not considering variables in the model to valid constraints:

### Not considering variables

By design, the proposed formulation does not consider some variables. Computationally, these variables are not even created to produce a more efficient formulation.

The variables that are not defined are:

- $u_{cp}\;\forall \;f\in F,\;c\notin CP_{f},\;p\in P$: patterns that cannot be assigned to certain clients. (PP1)
- $x_{ij}^{h}\;\forall \;h\in H,\;i\in C,\;j\in C\text{ if }a_{i}+s_{i}+t_{ij}>b_{j}-s_{j}$: arc (*i*,*j*) cannot be used if it is impossible to meet the time window’s requirement of client *j*. Note that $a_{i}+s_{i}+t_{ij}$ is the earliest possible arrival from *i* to *j* and $b_{j}-s_{j}$ is the later *j* can be visited. (PP2)

### Pattern assignation to avoid symmetry

Pattern assignation is decided in the model using variable *u*_*cp*_. For clients on *CP*_6_ that must be visited daily, there is only one pattern available. But for other clients, multiple patterns are available as presented in Table 1.

**Table 1:** Possible patterns according to *f.*

*f*/*p*	2	3	4	5	6	7	8	9	10	11	12
3	✓	✓									
2			✓	✓	✓						
1						✓	✓	✓	✓	✓	✓

It is important to remark that patterns are very symmetric because a condition of the enterprise is that the visits to each client are equally spaced in the planning horizon. This can be seen in Table 2. Considering that the other conditions of the problem (time windows, demands, times to traverse arcs, etc.) are not day-dependent, given a set of customers, attending all of them represents the same routing problem independently of the day chosen to serve them.

**Table 2:** Visits distribution according to pattern *p.*

	Monday	Tuesday	Wednesday	Thursday	Friday	Saturday
*p* = 1	✓	✓	✓	✓	✓	✓
*p* = 2	✓		✓		✓	
*p* = 3		✓		✓		✓
*p* = 4	✓			✓		
*p* = 5		✓			✓	
*p* = 6			✓			✓
*p* = 7	✓					
*p* = 8		✓				
*p* = 9			✓			
*p* = 10				✓		
*p* = 11					✓	
*p* = 12						✓

Let us illustrate this concept with a particular example. Suppose a client distribution where the client 1 has *f* = 6, clients 2 and 3 have *f* = 3, clients 4–6 have *f* = 2, and client 7 has *f* = 1. A possible pattern distribution to attend the aforementioned clients can be seen in Table 3 where the last column represents the value of the variable *u*_*cp*_ for each client supposing day 1 is Monday. But this is not an obligation as a company can start planning their routes on a Tuesday. So if day 1 is Tuesday the solution would be the same (remember that the routing is not day-dependent) but the values of *u*_*cp*_ change. Now $u_{1,1}=u_{2,3}=u_{3,2}=u_{4,6}=u_{5,4}=u_{6,5}=u_{7,8}=1$. With the same logic, day 1 could be Wednesday, Thursday, Friday or Saturday (only the values of *u*_*cp*_ change). As a consequence, there are six symmetric solutions that would bare the same result.

**Table 3:** Visits distribution of a given instance.

Client/day	1	2	3	4	5	6	*u*_*cp*_
1	✓	✓	✓	✓	✓	✓	*u*_1,1_ = 1
2	✓		✓		✓		*u*_2,2_ = 1
3		✓		✓		✓	*u*_3,3_ = 1
4	✓			✓			*u*_4,4_ = 1
5		✓			✓		*u*_5,5_ = 1
6			✓			✓	*u*_6,6_ = 1
7	✓						*u*_7,7_ = 1

To eliminate this symmetry, client seven could be forced to be served according to a given pattern (in this case *u*_7,7_ = 1). To generalize this assumption and reduce symmetry in the problem, the following equation is proposed (let us suppose *n*_1_ is the first client with frequency 1).

$$u_{n_{1},7}=1$$ (28)

This logic could also be applied to the first client with frequency two (*n*_2_), and the first client with frequency three (*n*_3_). Let us present two other assignment equations:

$$u_{n_{2},4}=1$$ (29)

$$u_{n_{3},2}=1$$ (30)

Note that these equations cannot be used at the same time as this can eliminate more feasible solutions apart of the symmetrical ones (and even optimal ones)

### Valid inequalities

Variables *u*_*cp*_ can be bounded considering the total possible demand the available vehicles can serve in a day. This can be done by comparing the demand served each day due to the assignment of *u*_*cp*_ to every client, with the aggregated capacity of the vehicles. The following constraints must be satisfied in order for the vehicles to meet daily demand.


$$\sum_{k\in K}q_{k}\geq \sum_{c\in C}dem_{c}\sum_{f\in F}\sum_{p\in P_{f}}A_{p}^{h}\cdot u_{cp},\;\forall \;h\in H$$

We can transform this into a valid inequality for a subset $C^{\prime }$ of the total of clients each one with a given pattern *p*_*c*_:

$$\sum_{c\in C^{\prime }}u_{cp_{c}}\leq |C^{\prime }|-1,\;\text{ if}\sum_{k\in K}q_{k}<\sum_{c\in C^{\prime }}A_{p_{c}}^{h}\cdot dem_{c}\;\forall \;h\in H$$ (31)

Another exploitable characteristic of the problem is related with the vehicles’ capacity and the clients’ demand. Let us consider an example: in the dataset presented in [2] (https://github.com/aarenas2/MDHFPCVRP-TW) the vehicles’s capacities are 12 (small) and 16 (medium). If client 1 has a demand of 10 vending machines, and client 2 a demand of 7 vending machines, this clients cannot be attended with the same vehicle. As a consequence, on any given day *h*, $x_{1,2}^{h}\;+\;x_{2,1}^{h}\leq 0$. If we group three clients (1, 2 and 3) instead of two, and the sum of the clients’ demands is greater than 16, now we have $x(\{1,2,3\}){\;\;}^{h}\leq 1$. This inequality can be interpreted as: the maximum number of active arcs in the subset of clients is one (more than one implies that the same vehicle visited all the nodes which is infeasible). Let us generalize this valid inequality for any set of clients $C^{\prime }$ as:

$$x(C^{\prime }{)}^{h}\leq |C^{\prime }|-2,\;\forall \;h\in H,\text{ if}\sum_{c\in C^{\prime }}dem_{c}>q^{m}$$ (32)

## Numerical results

This section is divided into two subsections: in the first one, the effects of the valid constraints, and preprocessing techniques are presented over a subset of instances. Then, with the best setup encountered, the performance of the 3IFv2 is evaluated versus the original formulations presented in [2]. All the experiments were run on a computer with an Intel Core i7 4 GHz processor with 64 GB RAM and Ubuntu as the operating system. The modeling was done in Python and solved using GUROBI 11.0.3. All the solver’s default parameters were used.

### Effects of valid constraints and preprocessing on linear relaxation

In this subsection, the effect of preprocessing techniques PP1 and PP2, and constraints (28) to (32) over the linear relaxation of the proposed model are evaluated. To do so, all the datasets used in [2] with total number of visits Ξ=120 (comprising 15 datasets) as those were the larger instances. The performance is measured in the average GAP from the best known value of the linear relaxation for each setup, and in the number of times each setup resulted in the best known linear relaxation. As (31) and (32) can be done with multiple sets of clients, for (31) we used every possible combination of three clients, meanwhile, for (32) we used every possible combination of three clients ((32)A) and four clients ((32)B). Let us present the results in Table 4 where BKN represents the number of times the best known linear relaxation was found using the technique on the column.

**Table 4:** Results of the preprocessing techniques and valid constraints over the linear relaxation of the 3IFv2.

	Original	PP1	PP2	(28)	(29)	(30)	(31)	(32)A	(32)B
Average	4.87%	4.87%	3.93%	4.80%	4.78%	4.82%	4.87%	4.48%	0.14%
BKN	0	0	2	0	0	0	0	0	13

The results in Table 4 demonstrate the impact of different preprocessing techniques and valid constraints on the model’s linear relaxation performance. The inequality using combinations of four clients (32)B proved most effective, achieving a 0.14% average GAP and finding the best known solution in 13 out of 15 instances. PP2 showed moderate effectiveness with a 3.93% average GAP and found the best solution twice, while using combinations of three clients (32)A achieved a 4.48% average GAP. PP1 provided no improvement over the original formulation, both showing 4.87% GAP. This could be due to constraints (3) already forcing those variables to zero. Nevertheless, PP1 is useful reducing the number of considered binary variables in the model. Pattern assignment constraints ((28), (29), (30)) and client combination constraints (31) delivered minimal improvements, with GAPs ranging from 4.78% to 4.87%. These results indicate that capacity-based inequalities incorporating larger client combinations were most successful at tightening the linear relaxation bounds, while simpler preprocessing and symmetry-breaking constraints demonstrated limited effectiveness in improving the model’s performance.

The final formulation used in the following section was the original 3IFv2 ((1) to (27)) plus:

- PP1 and PP2: even though did not improve much the linear relaxation, they reduce the number of binary variables.
- (29): eliminated some symmetry and had the best improvement on the linear relaxation of the pattern assignment constraints.
- (32)B: produced the tighter lower bounds.

### Results of the 3IFv2 versus previous formulations

Following the experimental methodology from [2], we evaluated the performance of 3IFv2 against the 4IF and 3IF formulations. The experiments were conducted on the same set of instances, grouped by total visits required (Ξ=30,60,90 and 120) and client frequency distribution (dense, balanced, and sparse). Each configuration was tested with five different randomly generated instances using identical parameters, with a 3600-second time limit. Results can be seen in Tables 5 to 8. The code to generate the instances can be found in https://github.com/aarenas2/MDHFPCVRP-TW).

**Table 5:** Results with Ξ=30.

Parameters		Avg. GAP (%)			Avg. TFFFS (s)			NFS		
Distribution	Clients	4IF	3IF	3IFv2	4IF	3IF	3IFv2	4IF	3IF	3IFv2
Dense	8	0.6%^(4)^	0.0%^(5)^	0.0%^(5)^	1.0	1.0	1.0	0/5	0/5	0/5
Balanced	11	2.0%^(2)^	1.1%^(3)^	0.1%^(4)^	11.4	1.6	3.4	0/5	0/5	0/5
Sparse	13	3.2%	1.5%^(3)^	0.0%^(5)^	24.8	4.0	10	0/5	0/5	0/5

**Table 6:** Results with Ξ=60.

Parameters		Avg. GAP (%)			Avg. TFFFS (s)			NFS		
Distribution	Clients	4IF	3IF	3IFv2	4IF	3IF	3IFv2	4IF	3IF	3IFv2
Dense	16	3.8%	4.8%	0.7%^(1)^	61.0	30.2	5.8	0/5	0/5	0/5
Balanced	22	6.1%	5.1%	2.1%	73.4	79.8	19.0	0/5	0/5	0/5
Sparse	26	9.5%	12.3%	4.4%	486.0	164.2	75.4	0/5	0/5	0/5

**Table 7:** Results with Ξ=90.

Parameters		Avg. GAP (%)			Avg. TFFFS (s)			NFS		
Distribution	Clients	4IF	3IF	3IFv2	4IF	3IF	3IFv2	4IF	3IF	3IFv2
Dense	24	6.4%	9.3%	2.9%	430.6	152.6	23.2	0/5	0/5	0/5
Balanced	33	10.0%	10.6%	4.5%	2805.0	850.6	124.0	4/5	0/5	0/5
Sparse	39	17.4%	13.5%	7.0%	1605.5	812.6	87.8	3/5	0/5	0/5

**Table 8:** Results with Ξ=120.

Parameters		Avg. GAP (%)			Avg. TFFFS (s)			NFS		
Distribution	Clients	4IF	3IF	3IFv2	4IF	3IF	3IFv2	4IF	3IF	3IFv2
Dense	32	7.8%^*a*^	7.2%	3.5%	1766.0	742.4	81.2	4/5	0/5	0/5
Balanced	44	-	15.0%	6.6%^*b*^	-	1774.7	554.0^*b*^	5/5	2/5	0/5
Sparse	52	-	14.8%	8.6%	-	78.5	1687.7	5/5	3/5^*c*^	2/5^*c*^

^*a*^ Computed using only the instance where the 4IF was able to find a feasible solution.

^*b*^ Computed using only the instances where the 3IF was able to find a feasible solutions.

^*c*^ The two instances where the 3IF was able to find a feasible solution were the two instances where the 3IFv2 was not able to find any solution.

The results demonstrate that 3IFv2 consistently outperforms both previous formulations across all instance sizes and distributions. For small instances (Ξ=30), 3IFv2 achieves optimality in almost all cases (number in parenthesis next to Avg. GAP), with average GAPs of 0% for both dense and sparse distributions, and 0.1% for balanced instances. The time to find first feasible solutions (TFFFS) remains competitive with 3IF while significantly improving upon 4IF.

As instance size increases, 3IFv2’s advantages become more pronounced. For Ξ=60, it maintains single-digit GAPs (0.7%-4.4%) while both 4IF and 3IF show deteriorating performance (3.8%-12.3%). The TFFFS values for 3IFv2 are consistently lower, often by an order of magnitude compared to the other formulations.

The most striking improvements appear in large instances (Ξ=90,120). For Ξ=120, while 4IF fails to find solutions in most cases and 3IF struggles with feasibility (2-3 failures out of 5), 3IFv2 finds solutions in nearly all instances. The GAPs for 3IFv2 remain reasonable (3.5%-8.6%) compared to 3IF’s 7.2%-15.0%. Most notably, 3IFv2’s TFFFS values stay manageable (81.2-1687.7 seconds) when both other formulations frequently exhaust the time limit.

Finally, the fact that the 3IFv2 was able to find a feasible solution in 58 of 60 datasets demonstrates the strength of the formulation.

The consistent feasibility, lower GAPs, and faster solution times across all instance sizes demonstrate that 3IFv2’s structural improvements and valid constraints effectively strengthen the formulation while maintaining computational tractability.

Unfortunately, in the instance *MedellinVending262*, the 3IFv2 was not able to find a feasible solution after 12 hours. On the other hand from the 3IF and 4IF which exhausted the local memory of the CPU, the 3IFv2 improved the lower bound until time-limit was reached.

## Conclusions

We introduced a novel approach by replacing binary depot-client assignment variables with continuous auxiliary variables and using depot replication. This strategy simplifies the model structure while preserving its ability to capture complex routing decisions. The transformation in variable representation constitutes a significant advancement in modeling periodic vehicle routing problems with multiple attributes.

The preprocessing techniques and valid constraints demonstrated substantial impact in strengthening the model. PP2 improved the linear relaxation to 3.93% GAP with best known linear relaxation, while inequality (32) using combinations of four clients achieved a remarkable 0.14% average GAP. These enhancements significantly tightened the formulation’s bounds without compromising computational tractability or solution feasibility.

Computational experiments conclusively showed 3IFv2’s superiority over previous formulations. The new formulation achieved optimality for small instances, maintained single-digit relavite GAPs (0.7%-4.4%) for medium instances where 4IF and 3IF showed 3.8%-12.3%, and found feasible solutions for large instances where other formulations failed. Solution times improved by an order of magnitude, demonstrating 3IFv2’s enhanced computational efficiency.

For future work, we propose developing a matheuristic algorithm to solve large-scale instances like *MedellinVending262*. This approach would combine 3IFv2’s strong linear relaxation bounds with metaheuristic search procedures. The improved lower bounds from our formulation could guide the local search process while maintaining solution quality guarantees. This hybrid approach promises to extend the practical applicability of our model to industrial-scale problems.

We propose two directions for future research that may be relevant for the operational realities of vending companies: first, incorporating preventive maintenance scheduling for vending machines as an additional operational condition within the routing optimization; and second, implementing workload balancing constraints to ensure an equitable distribution of tasks among distributors or drivers.
